# Trends in Prevalence and Related Risk Factors of Overweight and Obesity among Women of Reproductive Age in Zimbabwe, 2005–2015

**DOI:** 10.3390/ijerph16152758

**Published:** 2019-08-02

**Authors:** Fadzai Mukora-Mutseyekwa, Hajo Zeeb, Lydia Nengomasha, Nicholas Kofi Adjei

**Affiliations:** 1 Clinical Research Centre, Africa University, Mutare, Zimbabwe; 2 Health Sciences Bremen, University of Bremen, 28359 Bremen, Germany; 3 Leibniz Institute for Prevention Research & Epidemiology-BIPS, D-28359 Bremen, Germany; 4 Department of Health Sciences, Africa University, Mutare, Zimbabwe

**Keywords:** obesity, overweight, determinants, trends, non-communicable diseases, Zimbabwe

## Abstract

*Background*: The prevalence of non-communicable diseases is rising in low and middle-income countries (LMICs) such as Zimbabwe, yet, the risk factors associated with overweight and obesity among women in the country have not been explored. This study investigated the trends in prevalence and demographic, socioeconomic and behavioral risk factors of overweight and obesity among Zimbabwean women of reproductive age (15–49 years) from 2005–2015. *Methods*: Data from the 2005/2006, 2010/2011 and 2015 Zimbabwe Demographic and Health Survey (ZDHS) were analyzed. Multiple logistic regression models were used to examine the associations between demographic, socioeconomic, behavioral risk factors and obesity and overweight (body mass index (BMI) ≥ 25.0 kg/m^2^). We further estimated the prevalence of overweight and obesity over the period covered by the surveys. *Results:* The prevalence of overweight and obesity increased substantially from 25.0% in 2005 to 36.6% in 2015. Some of the risk factors for overweight and obesity were older age (40+) (adjusted odds ratio (aOR) = 4.73; 95% confidence interval (CI) = 3.73–6.01) in 2015, being married, high economic status, being employed, residence in urban areas and alcohol use. Educational attainment and smoking status were not associated with overweight and obesity across all surveys. *Conclusions*: We provide the first detailed analysis of trends and risk factors for overweight and obesity between 2005 and 2015 among women in Zimbabwe. The findings indicate that women of reproductive age are at high, and increasing, risk of excess weight. Thus, prevention and control measures are needed to address the high prevalence of overweight and obesity in Zimbabwe.

## 1. Introduction

Obesity is associated with an increased risk for various important noncommunicable diseases (NCDs) such as diabetes, certain types of cancer and cardiovascular diseases (CVD) like hypertension, coronary heart disease, and stroke [1]. The World Health Organization (WHO) defines a person to be overweight if their body mass index (BMI) is > 25 kg/m^2^, and the condition is described as obesity if BMI is ≥ 30 kg/m^2^ [2]. It has been estimated that overweight and obesity contributed to 3.4 million global annual deaths, 3.9% years of life lost and 3.8% of global disability-adjusted life years (DALYs) in 2010 [3,4].

Worldwide prevalence of obesity was noted to have tripled between 1975 and 2016 with women being particularly affected [5]. Among women globally, a significant increase in the prevalence of excess weight has been observed over a relatively short period, with the proportion of women with overweight and obesity increasing from 29.8% to 38% between 1980 and 2013 [6]. In Sub-Saharan African (SSA) in particular, prevalence of obesity and overweight have also been increasing at an alarming pace [7]. A 2015 review of obesity in SSA shows that while overweight and obesity rates are increasing in all African regions, Southern Africa is most affected [8]. A recent analysis of Demographic and Health Survey (DHS) data from 32 SSA countries revealed a pooled prevalence of 15.9% overweight, ranging from 5.6% in Madagascar to 27.7% in Swaziland. Obesity ranged from 1.1% in Madagascar to 23% in Swaziland [9].

In developing countries, an association has been found between socioeconomic inequalities and the risk factors for NCDs such as obesity [10]. In the SSA 32-country DHS analysis, wealth index (rich vs. poor) was the strongest predictor for overweight and obesity among women in most of the countries [9]. Other respective demographic factors that have been implicated as key determinants of overweight and obesity in other studies in LMICs include urban residence, high education and older age [9,11,12,13,14]. Of note is the observation that these socioeconomic status associations of overweight/obesity are different as compared to some Western high-income countries where overweight and obesity are generally concentrated in the lower socioeconomic strata [15,16].

Prior literature shows that socioeconomic status, age, parity, marital status, physical inactivity, bodyweight perceptions and increased energy intake are factors highly predictive of excess weight in SSA [8,17]. Results from epidemiologic data have shown a mixed relationship between excessive body weight and other lifestyle factors, such as alcohol consumption or smoking [18,19,20,21,22]. 

Even though sound epidemiologic data are scarce, NCDs are recognized to be an important public health issue in Zimbabwe, coming second on the prioritization list in the current National Health Strategy (2016–2020) [18]. In terms of implementation, however, limited effort has been invested towards addressing overweight and obesity or its risk factors. This study aimed to describe the trends in the prevalence of overweight and obesity among Zimbabwean women aged 15 to 49. The investigation of prevalence and trends is crucial to inform advocacy efforts on the need for political prioritization of interventions aimed at mitigation of risk factor exposure and enabling lifestyle modification. This study also sought to explore the social, demographic, economic and lifestyle risk factors of overweight and obesity in this population, as this has not been explored nor documented in Zimbabwe at the scale made possible by the DHS dataset so far. Findings should provide evidence to direct policy-makers and implementers to tailor their interventions for this public health problem towards the appropriate socio-demographic groups.

## 2. Materials and Methods

### 2.1. Data

The data used in the present study were derived from the 2005/2006, 2010/2011 and 2015 Zimbabwe Demographic and Health Surveys (ZDHS). These surveys were undertaken by the Zimbabwe National Statistical Agency, and they were nationally representative surveys of men and women in their reproductive age. The surveys used a two-stage stratified cluster sampling method based on census enumeration areas (EAs) and household samples. The first stage was the selection of EAs with probability proportional to the size. The second stage involved household selection, where households were selected based on the EAs. The analysis was limited to non-pregnant women aged 15–49 years with valid weight and height measurements (survey year: 2005/2006; *n* = 8185), (survey year: 2010/2011; *n* = 8448) and (survey year: 2015; *n* = 9066). Including pregnant women in the analysis may present a misleading picture about the issue of overweight and obesity among women since they naturally gain weight during their pregnancy.

### 2.2. Measurement of Outcome Variable

The outcome variable was overweight and obesity. This variable was derived from the body mass index data of non-pregnant women in the various surveys. The body mass index (BMI; weight (kg)/height (m) squared) is a widely used measure for defining overweight and obesity [2]. According to the WHO standard cut-offs, a BMI of 25.0–29.9 kg/m^2^ is classified as overweight, and a BMI ≥ 30.0 kg/m^2^ is classified as obesity [2]. Overweight and obesity were combined as one category to ensure enough cases for the analysis. Women with a BMI of 25.0 kg/m^2^ or above were categorized as individuals with overweight and obesity and coded “1” while those below 25.0 kg/m^2^ were categorized otherwise and coded “0”. Anthropometric measurements were taken using calibrated equipment. Weight measurements were taken using the United Nations Children’s Fund (UNICEF) electronic scale with a digital display and height measurements were taken using a measuring board.

### 2.3. Independent Variable

The independent variables used in this study were categorized into three groups: socioeconomic status (SES), demographic factors and behavioral factors. SES was measured using three indicators: wealth (poorest, poorer, middle and richer), educational level (no formal education, primary, secondary or higher education) and employment status (currently employed, not currently employed). The demographic factors included age (15–19, 20–24, 25–29, 30–34, 35–39, 40+) and marital status (never married, currently married, living together, widowed, divorced or separated). Tobacco smoking and alcohol consumption were the two behavioral factors considered. Currently smoking (yes or no) was defined as smoking at least one cigarette or any form of tobacco a day over the past 30 days. Currently drinking (yes or no) was also defined as consuming one or more bottles of an alcoholic drink in a week. Other explanatory variables included the place of residence (rural or urban) and region or province. Administratively, Zimbabwe has been divided into ten regions or provinces. All the variables were obtained from two types of questionnaires: the individual women’s questionnaire and the household questionnaire. The individual women’s questionnaire provided information on the women (i.e., demographic, socioeconomic and lifestyle characteristics) while the household questionnaire provided information on household possessions and amenities such as sanitation facilities, source of drinking water and household’s ownership of selected assets, which were used to create the “wealth index”.

### 2.4. Statistical Analysis

Both descriptive and regression analyses were performed in this study. The first part of the analysis was primarily descriptive, where prevalence and trends of overweight and obesity were calculated. Differences in obesity and overweight prevalence rates between the three survey years were examined using the chi-squared test. In the second part, binary logistic regression models were fitted to examine the associations between the independent variables and the outcome variable. The binary logistic models estimate the probability of the outcome variable (overweight and obesity) to be 1 (*h* = 1). More formally, the conditional probability of experiencing the event (overweight and obesity) can be expressed as:

$$pr(h=1|x)=\frac{\exp(x\beta )}{1+\exp(x\beta )}$$

Prevalence and odds ratios with 95% confidence intervals (95% CI) were calculated using Stata Version 14 (Stata Corp, College Station, TX, USA).

### 2.5. Ethical Considerations

This study involved secondary analysis of a pre-existing dataset obtained through a standard national survey. The authors were granted approval to obtain and use the collected data for analysis from the Demographic and Health Survey (DHS) review board. All data were anonymized prior to the authors receiving the data.

## 3. Results

### 3.1. Distribution of Characteristics 

Results from Table 1 show the distribution of respondents’ characteristics. The mean age of respondents was similar across all surveys, approximately 28 years. Meanwhile, the mean BMI values increased from 23.17 kg/m^2^ in 2005 to 26.89 kg/m^2^ in 2010, and then decreased to 24.56 kg/m^2^ in 2015.

### 3.2. Trends Over Time in the Prevalence of Overweight and Obesity

The prevalence of overweight and obesity (BMI ≥ 25.0 kg/m^2^) by socioeconomic status (SES), demographic and behavioral factors is shown in Table 2.

The prevalence of overweight and obesity increased substantially from 25.0% in 2005 to 36.6% in 2015. Age showed a marked and continuous increase in the trend of the prevalence of overweight and obesity across all surveys. While the highest increase from 2005 to 2015 was among those who were 35 years and above, the prevalence decreased slightly from 15.5% in 2010 to 13.7% in 2015 for the 15–19 years age groups. Regarding marital status, while the prevalence remains relatively unchanged among never-married women between 2010 (20.2%) and 2015 (19.3%), it increased remarkably among married and cohabiting women over the same period from 37.9% to 44.3%.

**Figure 1:**
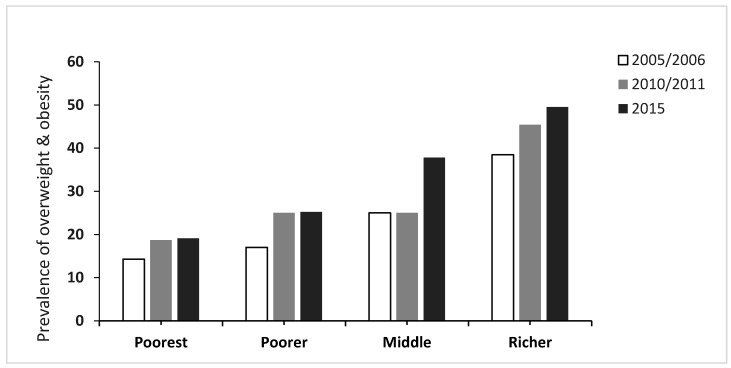
Prevalence of overweight and obesity by wealth index among women of reproductive age (15–49 years), Zimbabwe, 2005–2015.

Similar observations were made when stratifying data by socioeconomic status (SES) of women. Although the prevalence of overweight and obesity was relatively lower among the poorest, we observed a slight increase in the prevalence among this sub-group from 14.3% in 2005 to 19.1% in 2015. Meanwhile, the prevalence of overweight and obesity among the rich increased considerably from 38.5% in 2005 to 49.5% in 2015 (Figure 1). Considering employment status, the prevalence of overweight and obesity was higher among women who were employed. While the prevalence of overweight and obesity showed a substantial increase from 41.0% in 2010 to 46.9% in 2015 among those who were currently employed, it basically remained stable (2010: 28.6%; 2015: 29.3%) among those who were not employed. Regarding educational attainment, we observed that the prevalence of overweight and obesity was fairly similar across all educational levels and the trends did not show a consistent pattern across the sub-groups.

The trend analysis also showed a consistent increase in the prevalence of overweight and obesity over time for the behavioral factors. Current smokers had the highest prevalence of overweight and obesity during the entire period (2005–2015). The prevalence increased substantially from 22.1% in 2005 to 51.0% in 2015. Current drinkers of alcohol had the highest prevalence of overweight and obesity in 2015 (55.3%). However, due to the unavailability of data for alcohol consumption before 2015, we could not examine trends for those consuming alcohol.

An exploration with respect to place of residence showed that women in the urban areas had the highest prevalence. However, we observed an increase in prevalence of overweight and obesity among women who live in the rural areas from 18.9% in 2005 to 28.5% in 2015. Nevertheless, the prevalence of overweight and obesity showed a striking geographical pattern where overweight and obesity were most frequent in provinces or regions that are mostly urban. For instance, provinces such as Harare, Bulawayo and Manicaland had the highest prevalence of overweight and obesity, and it increased gradually over the period covered by these surveys.

### 3.3. Logistic Regression

The results of the adjusted OR and 95% CI for the relationship between demographic factors, socioeconomic status, behavioral factors and overweight and obesity are shown in Table 3.

### 3.4. Relationship between Socioeconomic Status and Demographic Factors with Overweight and Obesity

Results from Table 3 revealed that wealth and employment status were the two important socioeconomic determinants of overweight and obesity. Interestingly, educational attainment was not associated with overweight and obesity across all surveys. The wealth index showed a positive association with overweight and obesity and a consistent gradient was found. Richer women had higher odds of being overweight and obese (aOR = 3.11; 95% CI = 2.40–4.10) for 2005, (aOR = 3.06; 95% CI = 2.47–3.78) for 2010 and (aOR = 3.61; 95% CI = 2.89–4.51) for 2015 compared to the poorest. We also observed a strong association between employment status and overweight and obesity. Women who were employed were slightly more likely to be overweight and obese (aOR = 1.27; 95% CI = 1.13–1.42) for 2005 and (aOR = 1.19; 95% CI = 1.07–1.32) for 2015 compared to those who were not employed.

Regarding demographic factors, Table 2 shows that age is another important determinant of overweight and obesity. Older women (40+) years were more than five times likely to be overweight and obese (e.g., aOR = 4.73 (95% CI = 3.73–6.01) in 2015 compared to younger women (15–19) years). We also found marital status to be strongly associated with overweight and obesity, where married women had higher odds of overweight and obesity at all survey waves (aOR ranging from 1.32 to 1.72) compared to never married women.

The regression analysis also confirmed the role of geographical area or location. Women living in the rural areas were about 30% less likely to be overweight and obese than their counterparts in urban areas. For instance, those from the largely rural Mashonaland West province were less likely to have overweight and obesity (aOR = 0.81; 95% CI = 0.66–1.01) in 2015 than residents from the Manicaland Province.

### 3.5. Overweight and Obesity and Potential Modifiable Risk Factors

Smoking cigarettes or any form of tobacco had no significant association with the risk of overweight or obesity over the period covered by the surveys. Meanwhile, we found that non-consumers of alcoholic beverages had reduced odds of overweight and obesity (aOR = 0.48; 95% CI = 0.38–0.62) in 2015 compared to those who consumed alcoholic beverages

## 4. Discussion

According to the most recent ZDHS (2015), women in Zimbabwe exhibit a high prevalence of overweight and obesity (1 in every 3 women aged 15 to 49). The evidence over time has persistently shown that the burden of excess weight is higher among women than in men [6,19,20,21]. In Zimbabwe, the prevalence among women (35%) is considerably higher than their male counterparts at 12% [22]. In this study, we explored the trends in the prevalence of overweight and obesity among Zimbabwean women aged 15 to 49. We further examined the social, demographic, economic and lifestyle risk factors of overweight and obesity in this population.

Our study revealed a substantial 11.6% increase in the prevalence of overweight and obesity among women of reproductive age in Zimbabwe from 2005 to 2015. Several previous studies have also reported obesity and overweight to be on the rise in other developing countries [6,23,24]. The dire public health implication of these findings is the predictable risk of a high burden of obesity related morbidity and mortality in the future.

We found, older age, being married, higher wealth status, being employed and urban residence to be significantly associated with overweight and obesity; but no significant association could be established between excess weight and level of education and smoking. Notably, however, even among ‘lower-risk’ groups such as women of lower wealth status or those residents in rural areas, trends still showed a steady increase in the prevalence of overweight and obesity over time.

Our age-related findings are in line with the literature, which consistently show the prevalence of overweight and obesity to be higher in older women [14,25,26]. It has been advanced that as women grow older they tend to engage in less physical activity and consume a higher intake of energy [27].

Associations between marital status and overweight have been found in this and numerous other studies [14,17,26]. Married women are likely to have higher parity which may be linked to adopting a more sedentary lifestyle [26]. It is also suggested that women tend to be offered high energy foods during the postpartum period [26]. Other explanations are that while unmarried people devote more time to exercise and eat healthy [28], their married counterparts lack this motivation and spend more time on sedentary activities [17]. In Zimbabwe, cultural notions advance the expectation of weight gain among women following marriage, considered a sign of contentment in a happy union.

Women in a higher economic position were also seen to have higher odds of overweight or obesity. Although this finding is not in line with literature from some developed world settings where higher SES has been shown to be associated with a reduced risk of excess weight [15,16], it is consistent with studies conducted in other LMICs [9,14,26]. In the latter setting, these economic sub-groups tend to be more exposed to unhealthy lifestyle choices because they find access to energy-dense foods more affordable while also more likely to follow a sedentary lifestyle. Studies suggest that although wealthier women may be exposed to resources and education on healthy lifestyles, their knowledge may not automatically translate into practice because of several socio-cultural barriers [24,29]. Cultural norms biased towards fatter body size have been advanced as significantly contributing towards the observed excess weight SES differences in Africa [30].

Physical activity related to occupation is known to be protective against excess weight [31]. Employed women in our study had higher odds of overweight and obesity. In the 2005 Zimbabwe STEPS survey, 57% of employed women reported physical inactivity during working hours (defined as work involving mostly sitting or standing with walking for no more than 10 min at a time). Job characteristics such as irregular schedules, shift work, short breaks, lack of physical job demands and limited food options have been shown to be among the contributors to poor eating and exercise behaviors at the workplace [32]. Work environments for most employed Zimbabwean women tend to support inactive pursuits such as sitting at the marketplace and selling wares or office work.

Contrary to findings in most of the literature from similar settings, level of education did not emerge as an important factor for overweight and obesity among Zimbabwean women. We noted that this observation may be stirred on by the generally high literacy levels pegged at 88.2% among women aged 15+ according to 2014 data from the United Nations Educational Scientific and Cultural Organization (UNESCO) [33]. Elsewhere in similar settings, prevalence of overweight and obesity seems significantly higher among women who have attained at least a high school education and above [11,14,26] which is thought to be explained by the resultant shifts from manual labor to more sedentary occupations among the more educated [26]. Residence in urban areas also emerged as a key determinant of overweight and obesity among Zimbabwean women in this study. This is alarming given the ongoing urbanization of Zimbabwe in similar countries. It is projected that by 2020, half of the African population will be residing in urban environments [34], and the increasing level of urbanization in Africa, with its associated nutritional transition including increased access to fast food outlets [34], has been cited as one of the most important factors contributing to the emerging prevalence of overweight and obesity in the region [8,17].

Rural residents are more likely to access healthier, fresh and more natural food options [9]. It has also been suggested, however, that rural women are just unable to afford enough food due to raised food prices resulting from hostile economic environments and climate change in most developing countries [35,36].

A significant statistical association was found between alcohol consumption, overweight and obesity among Zimbabwean women of reproductive age. Although the evidence for this relationship is conflicting in different places, a relationship between heavy alcohol consumption and excessive weight has been found repeatedly [37,38]. In our analysis, smoking did not emerge as a predictor for overweight and obesity among Zimbabwean women aged 15 to 49, with an overall very low prevalence (1%) of smoking among women in Zimbabwe compared to 17% among men [22]. Other studies in countries with higher smoking frequencies among women showed current smokers had decreased odds of being overweight or obese compared to nonsmokers, however, among overweight/obese women, heavy daily smokers were the most vulnerable for abdominal obesity [39,40].

### Strengths and Limitations 

The major strength of this study was that nationally representative DHS data was used. The DHS survey employs standardized data collection protocols administered by trained study personnel with standardized measurement equipment using validated questionnaires. Nonetheless, some study limitations were also observed. Since secondary data was used, information on other important behavioral factors that could have explained the prevalence of excess weight in this population was not available. This includes diet (nutritional history) and physical inactivity. Data on alcohol consumption was only available for the most recent ZDHS (2015). Additionally, causality of associations cannot be established because of the cross-sectional methodology employed in the DHS. There was also no data on waist circumference, which would have allowed examination of trends in central obesity.

## 5. Conclusions

We provide the first evidence of trends and associations between risk factors and overweight and obesity among women of reproductive age in Zimbabwe between 2005 and 2015. The findings indicate that women of a reproductive age are at a high risk of obesity, with an alarming rate of increase in prevalence over the ten-year period under study. Long-term adverse health consequences are foreseeable if the trends remain uncurbed. 

The study revealed that among women of reproductive age, certain demographic characteristics are more likely to be associated with excess weight states such as those employed, higher socioeconomic strata, older age group, married and residing in urban areas. Public health interventions particularly targeting women with these identified risk factors with health education, lifestyle modification, weight reduction and maintenance strategies, as well as setting-based interventions are urgently needed to address the high prevalence of overweight and obesity in Zimbabwe.

## Figures and Tables

**Table 1:** Percentage distribution of the characteristics of women in Zimbabwe, 2005–2015.

Variables	2005/2006	2010/2011	2015
	(*n* = 8158)	(*n* = 8448)	(*n* = 9066)
	*N* (%)/Percentile	*N* (%)/Percentile	*N* (%)/Percentile
**Body Mass Index (BMI) kg/m^2^ (Mean (± SD))**	23.17 (5.20)	26.89 (16.01)	24.56 (5.53)
5th percentile	17.77	18.09	18.24
10th percentile	18.66	18.91	19.21
75th percentile	24.99	26.47	27.02
95th percentile	31.22	40.17	34.49
**Age (Mean (± SD))**	27.86 (9.54)	28.32 (9.49)	28.62 (9.39)
15–19	1959 (24.10)	1867 (22.10)	1994 (21.99)
20–24	1718 (21.06)	1604 (18.99)	1571 (17.33)
25–29	1290 (15.81)	1501 (17.77)	1482 (16.35)
30–34	1108 (13.58)	1180 (13.97)	1423 (15.70)
35–39	793 (9.72)	955 (11.30)	1114 (12.29)
40+	1290 (15.81)	1341 (15.87)	1482 (16.35)
**Marital Status**			
Never married	2369 (29.04)	2292 (27.13)	2540 (28.02)
Currently married	4380 (53.69)	4694 (55.56)	5047 (55.67)
Living together	120 (1.47)	226 (2.68)	268 (2.96)
Widowed	636 (7.80)	575 (6.81)	409 (4.51)
Divorced/separated	653 (8.00)	661 (7.82)	802 (8.85)
**Parity**			
<2	3812 (46.73)	3797 (44.95)	3914 (43.17)
2–3	2376 (29.12)	2787 (32.99)	3173 (35.00)
4–5	1165 (14.28)	1270 (15.03)	1487 (16.40)
6+	805 (9.87)	594 (7.03)	492 (5.43)
**Place of residence**			
Urban	2966 (36.36)	3208 (37.97)	4079 (44.99)
Rural	5192 (63.64)	5240 (62.03)	4987 (55.01)
**Educational Level**			
No education	364 (4.46)	212 (2.51)	92 (1.01)
Primary	2703 (33.13)	2412 (28.55)	2179 (24.03)
Secondary and higher	5091 (62.41)	5824 (68.94)	6795 (74.95)
**Employment Status**			
Not currently employed	5142 (63.03)	5439 (64.38)	5317 (58.65)
Currently employed	3016 (36.97)	3009 (35.62)	3749 (41.35)
**Wealth (Index)**			
Poorest	1472 (18.04)	1550 (18.35)	1359 (14.99)
Poorer	1458 (17.87)	1426 (16.88)	1327 (14.64)
Middle	3216 (39.42)	3361 (39.78)	3750 (41.36)
Richer	2012 (24.66)	2111 (24.99)	2630 (29.01)
**Region**			
Manicaland	955 (11.71)	921 (10.90)	925 (10.20)
Mashonaland Central	680 (8.34)	824 (10.00)	891 (9.83)
Mashonaland East	638 (7.82)	783 (9.27)	825 (9.10)
Mashonaland West	707 (8.67)	889 (10.52)	964 (10.63)
Matebeleland North	632 (7.75)	714 (8.45)	795 (8.77)
Matebeleland South	586 (7.18)	779 (9.22)	743 (8.20)
Midlands	1021 (12.52)	901 (10.67)	967 (10.67)
Masvingo	875 (10.73)	723 (8.56)	959 (10.58)
Harare	1268 (15.54)	1108 (13.12)	1109 (12.23)
Bulawayo	796 (9.76)	806 (9.54)	888 (9.79)
**Currently Smoking**			
Yes	77 (0.94)	52 (0.62)	49 (0.54)
No	8081 (99.06)	8396 (99.38)	9017 (99.46)
**Currently Drinking**			
Yes	-		338 (3.73)
No	-		8728 (96.27)

**Table 2:** Prevalence of overweight and obesity by demographic, socioeconomic and behavioral risk factors among women of reproductive age (15–49 years) in Zimbabwe, 2005–2015.

Variables	2005/2006 (*n* = 8158)	2010/2011 (*n* = 8448)	2015 (*n* = 9066)
	Overweight and Obese (%)	Overweight and Obese (%)	Overweight and Obese (%)
**Age**			
15–19	11.5	15.5	13.7
20–24	18.2	22.9	25.5
25–29	25.7	34.9	37.5
30–34	31.9	41.1	47.5
35–39	37.7	44.3	52.3
40+	39.7	52.2	52.3
**Marital Status**			
Never married	15.4	20.2	19.3
Currently married	28.8	37.9	44.3
Living together	35.0	32.3	44.3
Widowed	32.9	41.4	46.0
Divorced/separated	24.7	35.7	40.3
**Parity**			
<2	17.34	23.70	23.94
2–3	29.63	38.39	45.89
4–5	34.76	43.78	46.20
6+	33.29	44.11	47.76
**Place of residence**			
Urban	35.5	44.5	46.5
Rural	18.9	26.0	28.4
**Educational Level**			
No education	26.9	36.3	32.6
Primary	21.9	30.3	29.7
Secondary and higher	26.4	34.0	29.7
**Employment Status**			
Not currently employed	21.6	28.6	29.3
Currently employed	30.6	41.0	46.9
**Wealth (Index)**			
Poorest	14.3	18.7	19.1
Poorer	17.0	25.0	25.2
Middle	25.0	25.0	37.8
Richer	38.5	45.4	49.5
**Region**			
Manicaland	26.9	34.3	35.8
Mashonaland Central	14.7	26.0	33.3
Mashonaland East	22.1	28.2	33.3
Mashonaland West	20.5	29.6	33.2
Matebeleland North	16.1	24.7	30.2
Matebeleland South	24.6	26.3	31.8
Midlands	21.3	32.5	34.2
Masvingo	20.9	31.0	35.1
Harare	36.2	47.0	47.7
Bulawayo	36.2	44.0	45.2
**Currently Smoking**			
Yes	22.1	34.6	51.0
No	25.0	33.0	36.5
**Currently Drinking**			
Yes	-	-	55.3
No	-	-	35.8
**Total**	25.0 ^a^	33.0 ^a^	36.6 ^a^

Note: ^a^ values with the same superscripts are significantly different between the surveys at *p* < 0.05.

**Table 3:** Multivariate associations between demographic, socioeconomic, behavioral risk factors and overweight and obesity among women of reproductive age (15–49 years), Zimbabwe, 2005–2015.

Variables	2005/2006	2010/2011	2015
	aOR (95% CI)	aOR (95% CI)	aOR (95% CI)
**Age**			
15–19 (ref)			
20–24	1.38 (1.12–1.71) ***	1.27 (1.04–1.54) **	1.47 (1.21–1.78) ***
25–29	2.06 (1.61–2.63) ***	2.19 (1.77–2.70) ***	2.24 (1.81–2.77) ***
30–34	2.72 (2.09–3.54) ***	2.71 (2.15–3.41) ***	3.21 (2.56–4.02) ***
35–39	3.40 (2.57–4.51) ***	3.23 (2.54–4.13) ***	3.98 (3.13–5.06) ***
40+	4.37 (3.28–5.83) ***	4.70 (3.66–6.02) ***	4.73 (3.73–6.01) ***
**Marital Status**			
Never married (ref)			
Currently married	1.32 (1.08–1.61) ***	1.44(1.21–1.71) ***	1.72 (1.45–2.05) ***
Living together	1.63 (1.05–2.53) **	1.14 (0.81–1.60)	1.11 (0.81–1.53)
Widowed	1.05 (0.80–1.37)	1.02 (0.79–1.32)	1.24 (0.94–1.63) *
Divorced/separated	0.97 (0.75–1.26)	1.24 (0.98–1.56)	1.29 (1.04–1.60) **
**Parity**			
<2 (ref)			
2–3	1.21 (1.01–1.44) **	1.16 (1.01–1.35) *	1.26 (1.09–1.47) **
4–5	1.37 (1.10–1.71) ***	1.37 (1.14–1.68) ***	1.21 (1.00–1.46) **
6+	1.34 (1.03–1.74) **	1.38 (1.14–1.68) **	1.53 (1.18–1.98) ***
**Place of residence**			
Urban (ref)			
Rural	0.72 (0.58–0.88) **	0.63 (0.53–0.74) ***	0.78 (0.68–0.90) ***
**Educational Level**			
No education (ref)			
Primary	0.97 (0.74–1.27)	1.02 (0.74–1.39)	1.16 (0.72–1.87)
Secondary and higher	1.16 (0.86–1.54)	1.12 (0.82–1.54)	1.39 (0.86–2.24)
**Employment Status**			
Not currently employed (ref)			
Currently employed	1.27 (1.13–1.42) ***	1.09 (0.98–1.22)	1.19 (1.07–1.32) ***
**Wealth (Index)**			
Poorest (ref)			
Poorer	1.25 (1.01–1.54) *	1.57 (1.30–1.90) ***	1.47 (1.20–1.78) ***
Middle	1.80 (1.48–2.19) ***	2.19 (1.84–2.61) ***	2.40 (2.01–2.87) ***
Richer	3.11 (2.40–4.01) ***	3.06 (2.47–3.78) ***	3.61 (2.89–4.51) ***
**Region**			
Manicaland (ref)			
Mashonaland Central	0.49 (0.37–0.64) ***	0.67 (0.54–0.84) ***	0.93 (0.75–1.15)
Mashonaland East	0.69 (0.53–0.88) **	0.72 (0.58–0.90) **	0.86 (0.70–1.07)
Mashonaland West	0.61 (0.48–0.79) ***	0.75 (0.60–0.92) **	0.81 (0.66–1.01) *
Matebeleland North	0.69(0.51–0.91) **	0.84 (0.66–1.08)	0.94 (0.75–1.17)
Matebeleland South	0.97 (0.75–1.25)	0.82 (0.65–1.03)	0.86 (0.69–1.08)
Midlands	0.64 (0.51–0.81) ***	0.93 (0.75–1.15)	0.91 (0.74–1.13)
Masvingo	0.87 (0.69–1.09)	1.01 (0.81–1.27)	0.99 (0.80–1.22)
Harare	0.86 (0.68–1.10)	1.01(0.80–1.25)	1.14 (0.93–1.39)
Bulawayo	0.85 (0.65–1.10)	0.96 (0.76–1.21)	0.98 (0.78–1.21)
**Currently Smoking**			
Yes (ref)			
No	1.69 (0.94–3.01)	1.45 (0.78–2.68)	0.83 (0.44–1.53)
**Currently Drinking**			
Yes (ref)	-	-	
No	-	-	0.48 (0.38–0.62) ***
**Observations**	8158	8448	9066
**Pseudo R^2^**	0.1129	0.1160	0.1422
**Log Likelihood**	−4086.2314	−4736.0011	−5106.2441

Notes: aOR, adjusted odds ratio, *** *p* < 0.001, ** *p* < 0.01, * *p* < 0.5.
